# Ultrasonographic Prediction of Placental Invasion in Placenta Previa by Placenta Accreta Index

**DOI:** 10.3390/jcm12031090

**Published:** 2023-01-31

**Authors:** Keita Hasegawa, Satoru Ikenoue, Yuya Tanaka, Maki Oishi, Toyohide Endo, Yu Sato, Ryota Ishii, Yoshifumi Kasuga, Daigo Ochiai, Mamoru Tanaka

**Affiliations:** 1 Department of Obstetrics and Gynecology, Keio University School of Medicine, 35 Shinanomachi, Sinjuku, Tokyo 1608582, Japan; 2 Department of Clinical Trial and Clinical Epidemiology, University of Tsukuba Faculty of Medicine, 1-1-1 Tennodai, Tsukuba 305-8577, Japan; 3 Department of Obstetrics and Gynecology, Kitasato University School of Medicine, 1-15-1 Kitazato, Minami, Sagamihara 252-0375, Japan

**Keywords:** placenta accreta spectrum, placenta previa, ultrasonography, placenta accreta index

## Abstract

This study aimed to investigate the diagnostic accuracy of the placenta accreta index (PAI) for predicting placenta accreta spectrum (PAS) in women with placenta previa. We analyzed 33 pregnancies with placenta previa at Keio University Hospital. The PAI was assessed in the early third trimester, and PAS was diagnosed histologically or clinically defined as retained placenta after manual removal attempts. The PAI and incidence of PAS were analyzed. Ten women (30%) were diagnosed with PAS and had higher volumes of perioperative bleeding (*p* = 0.016), higher rate of requiring uterine artery embolization (*p* = 0.005), and peripartum hysterectomy (*p* = 0.0002) than women without PAS. A PAI > 2 was the most useful cut-off point for predicting PAS and was more sensitive than prediction values using traditional evaluation (history of cesarean section and placental location). Post-hoc analysis revealed a higher rate of previous history of cesarean delivery (30% vs. 4.4%, *p* = 0.038), severe placental lacunae (≥grade2) (70% vs. 8.7%, *p* = 0.0003), thin myometrial thickness (90% vs. 22%, *p* = 0.0003), anterior placenta (100% vs. 30%, *p* = 0.0002), and presence of bridging vessels (30% vs. 0%, *p* = 0.0059) in PAS women. PAI could help predict the outcomes of women with placenta previa with and without a history of cesarean delivery to reduce PAS-induced perinatal complications.

## 1. Introduction

Placenta accreta spectrum (PAS) is first suspected when placenta previa is identified because 9.3% of placenta previa cases are associated with PAS [1]. Although the mortality rate of women with PAS has improved from 6–7% [2] to 0.05% recently [3], PAS is related to an increased risk of perinatal complications and interventions, such as excessive peripartum bleeding requiring blood transfusion, uterine artery embolization, and peripartum hysterectomy. Therefore, predicting PAS in the antepartum period is crucial because it is a means to decrease maternal morbidity/mortality.

Ultrasonography is the mainstay of prenatal diagnosis and monitoring, as well as preoperative prediction of PAS, and has a high accuracy for prenatal diagnosis of invasive placentation in high-risk pregnancies [4]. Rac et al. [5] recently reported using the placenta accreta index (PAI) scored by ultrasonography for predicting PAS; however, validation and replication studies for PAI are limited. Additionally, a previous study on the use of PAI only recruited women with a history of cesarean delivery [5]. It is well known that women without a history of cesarean delivery also have an increased risk of the adherent placenta in case of placenta previa [1].

Therefore, we aimed to investigate and validate the clinical utility of the PAI to predict PAS in women with placenta previa both with and without a history of cesarean delivery.

## 2. Materials and Methods

This was a single-center retrospective study. The hospital records of 33 consecutive women with singleton pregnancies, diagnosed with placenta previa at Keio University Hospital from June 2017 to January 2021, were analyzed. Placenta previa was diagnosed using transvaginal ultrasonography and defined as the presence of a placenta that completely covered the internal cervical ostium. Excluded were multiple pregnancies and patients who were referred to our hospital after having delivered elsewhere. All the ultrasonography images of patients with placenta previa have been stored. Women for whom ultrasonography images were unavailable or inadequate to evaluate PAI retrospectively were excluded from the analysis.

Abdominal and vaginal ultrasonography were performed by obstetricians trained in ultrasonography in the early third trimester. The ultrasound images were reviewed by a single observer (K.H.), and a PAI score was assigned preoperatively for each woman. Table 1 shows the parameters of the PAI. The PAI is a composite of the following five parameters: previous history of cesarean delivery, placental lacunae, smallest myometrial thickness, placental location, and bridging vessels [5].

PAS was diagnosed histologically after hysterectomy or clinically defined as placenta retained after previous attempts of manual removal according to the FIGO classification [6]. Depends on the predicted severity of the PAS, and for improving the perinatal outcomes, we decided the delivery timing of women with placenta previa between 34 and 37 weeks of gestation [7]. However, when the patient entered labor or had massive vaginal bleeding, emergency cesarean delivery was performed even before 34 weeks of gestation. Blood loss was counted as intraoperative bleeding.

The student’s *t*-test or chi-square test was used to test differences between the groups. The estimates and Clopper–Pearson confidence intervals for sensitivity, specificity, positive predictive value, and negative predictive value for the prediction of PAS were calculated for each cut-off point of the PAI. Receiver operating characteristic curve (ROC) analysis was performed for PAS prediction using the PAI; the area under the curve (AUC) was calculated, and the cut-off value of the PAI was calculated by using the Youden index. All statistical analyses were performed using SAS, version 9.4 (SAS Institute, Cary, NC, USA). Two-sided *p*-values < 0.05 were considered to indicate statistical significance.

The study was approved by the Ethics Committee of Keio University School of Medicine (No. 20030107). As all information was anonymous in the institutional database, informed consent from each patient was not needed.

## 3. Results

Maternal characteristics and perinatal outcomes are summarized in Table 2. Of the 33 women with placenta previa, 10 (30%) were diagnosed with PAS, and 23 did not have PAS. The PAS group showed a significantly larger volume of perioperative bleeding and higher rates of uterine artery embolization and peripartum hysterectomy than the non-PAS group.

The ROC curve predicting PAS using the PAI showed an AUC of 0.974 (95% confidence interval [CI], 0.925–1.00). A PAI > 2 was indicated as the most useful cut-off point for PAS prediction, with a sensitivity of 0.900 (95% CI, 0.555–0.997); specificity, 0.957 (95% CI, 0.781–0.999); positive predictive value, 0.900 (95% CI, 0.555–0.997); negative predictive value, 0.957 (95% CI, 0.781–0.999) (Table 3). These values were higher than the prediction rate of PAS based on the traditionally evaluated information (history of cesarean delivery and anterior placental location: sensitivity, 0.300; specificity, 0.957; positive predictive value, 0.750; negative predictive value; 0.759). Seven (70%) out of 10 women with PAS had no previous cesarean delivery, all of whom had a PAI > 2. Of the seven women with PAS without a history of cesarean delivery, five (71%) were aged above 35, three (43%) received infertility treatments, and only one (14%) had a history of uterine artery embolization.

The post-hoc analysis of the five parameters of the PAI score revealed significantly higher rates of previous cesarean deliveries ≥ 2 (30% vs. 4.4%, *p* = 0.038), placental lacunae ≥ Grade 2 (70% vs. 8.7%, *p* = 0.0003), myometrial thickness ≤ 5 mm (90% vs. 22%, *p* = 0.0003), placenta adhering to the anterior wall of the uterus (100% vs. 30%, *p* = 0.0002), and presence of bridging vessels to the bladder (30% vs. 0%, *p* = 0.0059) in the PAS group than in the non-PAS group.

## 4. Discussion

As previously reported, our study replicated the finding that PAS is associated with an increased risk of perinatal complications and requiring uterine artery embolization. Moreover, the present study indicated the clinical utility and significance of the PAI to predict PAS preoperatively in women with placenta previa both with and without a previous history of cesarean delivery, whereas previous study applied PAI only for women with a history of cesarean delivery [5]. In particular, a PAI > 2 indicated a practical cut-off point to predict PAS in women with placenta previa.

As expected, in the present study, the PAS group showed a significantly increased number of perioperative complications, including a larger amount of perioperative bleeding, a higher rate of uterine artery embolization, and peripartum hysterectomy than the non-PAS group. Per previous reports, PAS is associated with a significantly higher risk of blood transfusion (46.9%) and peripartum hysterectomy (52.2%) [3,8,9] which is consistent with this study’s findings. The PAI assessment may be clinically important for women with suspected placental invasion to reduce perinatal complications and maternal mortality associated with PAS.

The present study revealed that the PAI has high diagnostic accuracy for PAS. In particular, a PAI > 2 could be a useful cut-off point to predict PAS. Rac et al. [5] did not present a cut-off point for the PAI, but used it to help with risk stratification and counseling. Meanwhile, the present study suggests that PAI > 2 is useful for predicting PAS in women with and without a previous history of cesarean delivery. Of the five parameters comprising the PAI evaluated in this study (history of cesarean delivery, presence of placental lacunae, smallest myometrial thickness, placental location, and presence of bridging vessels to the bladder), significant differences were identified in all parameters between the PAS and non-PAS groups. We also reported on several ultrasonographic parameters that are associated with PAS. The sensitivity of placental lacunae for identifying placenta accreta was reported as 75% [10]. The sensitivity and specificity of the loss of the clear zone for identifying placenta accreta were reported as 74.9% and 76.9%, respectively [10]. Another study showed that the sensitivity, specificity, and positive and negative predictive values of placenta accreta using ultrasound findings were 53.3%, 88.1%, 82.1%, and 64.8%, respectively [11]. The prediction parameters calculated in the present study using the PAI were greater than those calculated in previous reports. On this basis, the diagnostic accuracy of PAI for PAS could be superior to the single ultrasonographic parameter-based method.

Happe et al. validated the predictability of the PAI for PAS by using 79 PAS cases, but only for women who had a history of previous cesarean delivery [12]. In fact, prior cesarean delivery has a large influence on PAI scoring [5], and the higher prevalence of cesarean deliveries has led to an increased incidence of PAS [13]. However, it is well known that women diagnosed with placenta previa even without previous cesarean delivery have an increased risk of PAS [1]. Indeed, the present study included seven (70%) women with PAS without a history of cesarean delivery, all of whom presented with PAI >2 and increased risk of PAS. The present findings potentially expand the utility of PAI for PAS prediction in patients even without a previous history of cesarean delivery.

Magnetic resonance imaging (MRI) is another modality used to predict PAS and MRI findings have been reported to be useful to define the topography and area of placental invasion [14,15]. Berkley et al. [16] reported that the sensitivity of MRI is 80–85% and the specificity is 65–100%. Fiocchi et al. [17] reported that MRI has 100% sensitivity and 92.3% specificity for the prediction of PAS. However, MRI may also mislead the diagnosis of PAS using ultrasonography [18], and it is not cost-effective as a screening tool for PAS. In this study, we revealed similar sensitivity and specificity of the PAI as for MRI for predicting PAS, indicating that the PAI has a high rate of diagnostic accuracy and exclusive diagnosis. Given these results, predicting PAS using ultrasonography may be preferable to using MRI.

Our study and a previous study have demonstrated the diagnostic accuracy of PAS using the PAI. However, Rac et al. [12] reported that the PAI could not help predict the depth of placental invasion. Recently, machine learning models have been used to predict the clinical outcomes in women with placenta accreta spectrum [19]. Because the severity of PAS (e.g., depth of placental invasion) is associated with increased maternal morbidity [20], further investigations including machine learning method and serum biomarkers are warranted to predict the severity of perioperative complications (blood loss, uterine artery embolization, and hysterectomy).

In our study, there were several strengths and limitations. The first strength was that the PAI was scored preoperatively and reviewed by a single observer, which could avoid observation bias and interobserver differences. The second strength was that the effectiveness of other prediction methods had not been demonstrated. Maternal serum alpha-fetoprotein, free beta-human chorionic gonadotropin [21,22], antithrombin III, PAI-1, soluble Tie2, and soluble vascular endothelial growth factor receptor 2 have been shown as biomarkers to predict PAS [23]. In addition, the maternal serum VEGF and Serum Cripto-1 levels have been reported as novel biomarkers to predict abnormally invasive placenta [24,25]. These biomarkers might aid clinicians additionally to ultrasonography in detecting PAS cases in the early weeks of gestation. Meanwhile, the first limitation was a small sample size, which might affect the statistical power of the present results. In addition, women with PAS in the present study had risk factors besides a history of cesarean delivery. The second limitation of our study was that patients with PAS accounted for approximately 30% of all the placenta previa cases, which is higher than the general frequency [1]. This may be related to the fact that our institution is a tertiary center and that many of our patients are elderly or post-IVF pregnant women. The fact that our institution is a tertiary center also resulted in high rates of blood loss, blood transfusion and embolization in the non-PAS group despite 65% being nulliparas without PAS. The third limitation was that systematic bias may have occurred because the observer could not be blinded to the patients’ risk factors completely. The last limitation was that we performed uterine artery embolization to preserve the uterus on maternal request for PAS cases where the placenta was found to be invading the uterine wall at cesarean delivery, where the placenta was retained after attempts at manual removal. Hence, these PAS cases were diagnosed clinically, and there was a lack of pathological evaluation.

In conclusion, the present study confirmed the clinical significance of the PAI in predicting PAS preoperatively in women with placenta previa, regardless of prior history of cesarean delivery. In particular, a PAI >2 was found to be a valid cut-off point to predict PAS in women who had placenta previa with and without a previous history of cesarean delivery. Assigning a PAI score could be clinically important to avoid perinatal complications and reduce maternal mortality associated with PAS.

## Figures and Tables

**Table 1:** Clinical values of obstetric parameters for evaluating the placenta accreta index.

Obstetric Parameter	Value
≥2 cesarean delivery	3.0
Lacunae	
Grade 3	3.5
Grade 2	1.0
Sagittal smallest myometrial thickness	
≤1 mm	1.0
<1 but ≥3 mm	0.5
>3 but ≤5 mm	0.25
Anterior placenta previa	1.0
Bridging vessels to the bladder	0.5

**Table 2:** Maternal characteristics and perinatal outcomes.

	PAS *n* = 10	Non-PAS *n* = 23	*p*-Value
Maternal age, years	39 ± 3.3	38 ± 5.2	0.59
BMI, kg/m^2^	20 ± 3.0	22 ± 3.6	0.12
Nulliparas	4 (40%)	15 (65%)	0.17
Gestational age at delivery, weeks	35.2 ± 1.5	35.5 ± 2.4	0.51
Perioperative blood loss, g	2913 ± 1314	1650 ± 841	**0.01**
Uterine artery embolization	6 (60%)	3 (13%)	**<0.01**
Blood transfusion	9 (90%)	14 (61%)	0.09
Peripartum hysterectomy	5 (50%)	0 (0%)	**<0.01**
Birth weight, g	2372 ± 427	2333 ± 505	0.83
Apgar score at 1 min < 7	2 (20%)	7 (30%)	0.54
Apgar score at 5 min < 7	0 (0%)	3 (13%)	0.23

Continuous variables are presented as means ± standard deviations. Categorical variables are presented as *n* (%). Statistically significant *p*-values are shown in **bold** text. Abbreviations: BMI, body mass index, PAS, placental accreta spectrum.

**Table 3:** Sensitivity, specificity, and positive and negative predictive values corresponding to each PAI score.

PAI	Non-PAS	PAS	Sensitivity (95% CI)	Specificity (95% CI)	PPV (95% CI)	NPV (95% CI)
>0	9	10	100.0 [69.2–100.0]	60.9 [38.5–80.3]	52.6 [28.9–75.6]	100.0 [76.8–100.0]
≤0	14	0				
>1	5	10	100.0 [69.2–100.0]	78.3 [56.3–92.5]	66.7 [38.4–88.2]	100.0 [81.5–100.0]
≤1	18	0				
>2	1	9	90.0 [55.5–99.7]	95.7 [78.1–99.9]	90.0 [55.5–99.7]	95.7 [78.1–99.9]
≤2	22	1				
>3	1	5	50.0 [18.7–81.3]	95.7 [78.1–99.9]	83.3 [35.9–99.6]	81.5 [61.9–93.7]
≤3	22	5				
>4	1	5	50.0 [18.7–81.3]	95.7 [78.1–99.9]	83.3 [35.9–99.6]	81.5 [61.9–93.7]
≤4	22	5				
>5	0	2	20.0 [2.5–55.6]	100.0 [85.2–100.0]	100.0 [15.8–100.0]	74.2 [55.4–88.1]
≤5	23	8				

Values are presented as median (Interquartile range). Abbreviations: PAI, placenta accreta index, PAS, placental accreta spectrum, PPV, positive predictive value, NPV, negative predictive value, CI, confidence interval.

## Data Availability

The data presented in this study are available on reasonable request from the corresponding author.
